# An Enhanced Machine Learning Approach for Brain MRI Classification

**DOI:** 10.3390/diagnostics12112791

**Published:** 2022-11-14

**Authors:** Muhammad Hameed Siddiqi, Mohammad Azad, Yousef Alhwaiti

**Affiliations:** College of Computer and Information Sciences, Jouf University, Sakaka 2014, Aljouf, Saudi Arabia

**Keywords:** brain, MRI, medical imaging, feature extraction, recognition, healthcare

## Abstract

Magnetic Resonance Imaging (MRI) is a noninvasive technique used in medical imaging to diagnose a variety of disorders. The majority of previous systems performed well on MRI datasets with a small number of images, but their performance deteriorated when applied to large MRI datasets. Therefore, the objective is to develop a quick and trustworthy classification system that can sustain the best performance over a comprehensive MRI dataset. This paper presents a robust approach that has the ability to analyze and classify different types of brain diseases using MRI images. In this paper, global histogram equalization is utilized to remove unwanted details from the MRI images. After the picture has been enhanced, a symlet wavelet transform-based technique has been suggested that can extract the best features from the MRI images for feature extraction. On gray scale images, the suggested feature extraction approach is a compactly supported wavelet with the lowest asymmetry and the most vanishing moments for a given support width. Because the symlet wavelet can accommodate the orthogonal, biorthogonal, and reverse biorthogonal features of gray scale images, it delivers higher classification results. Following the extraction of the best feature, the linear discriminant analysis (LDA) is employed to minimize the feature space’s dimensions. The model was trained and evaluated using logistic regression, and it correctly classified several types of brain illnesses based on MRI pictures. To illustrate the importance of the proposed strategy, a standard dataset from Harvard Medical School and the Open Access Series of Imaging Studies (OASIS), which encompasses 24 different brain disorders (including normal), is used. The proposed technique achieved the best classification accuracy of 96.6% when measured against current cutting-edge systems.

## 1. Introduction

The brain, which is the human body’s most important structural element, contains 50–100 trillion neurons [1]. It is also known as the human body’s core section. Furthermore, it is known as the “processor” or “kernel” of the nervous system, and it plays the most important and critical role in the nervous system [2,3]. To the best of our knowledge, diagnosing brain disease is too difficult and complex due to the presence of the skull around it [4].

Utilizing technology to evaluate individuals with the aim of identifying, tracking, and treating medical issues is known as medical imaging. In medical imaging, magnetic resonance imaging (MRI) is a precise and noninvasive technique that can be used to diagnose a variety of disorders. In the last few decades, many scholars have proposed various state-of-the-art methods for brain MRI classification, and most of them focused on various modules of the MRI systems.

A latest convolutional neural network-based MRI method, data expansion, and image processing were proposed by [5] to recognize brain MRI images in various diseases. They compared the significance of their approach with pre-trained VGG-16 in the presence of transfer learning using a small dataset. Another deep learning-based method for detecting brain tumors in MRI images was created by [6]. This approach was divided into three phases: in the first phase, the CNN-based classifiers were implemented; while in the second phase, a region-based CNN was utilized on the output of the first phase; finally, in the third phase, the boundary of the brain tumor was focused and segmented by the Chan-Vese energy function followed by the edge detection method. On the other hand, an integrated approach was designed by [7], combining a mathematical morphological operator and an OASIS operator. In the first step, they extracted the largest connected area, such as the brain. After this, the unsupervised framework was employed to extract the various axial slices of brain. The main contribution of this paper was to identify the brains automatically, which was evaluated through five matrices using a publicly available dataset. Similarly, the glioma disease was analyzed by [8], where they utilized the Gaussian Naïve Bayes technique. In their approach, they employed the grow cut method followed by 3D features on MRI images. Then, they statistically analyzed the corresponding values through Spearman and Mann–Whitney U tests and achieved better results than the standard MRI dataset. The authors of [9] proposed an integrated approach for the detection of the tumor on brain MRI images. This approach is the combination of two well-known methods, morphological edge detection followed by fuzzy methods, respectively. In this method, the authors located the tumor through edge detection methods, while their performances were enhanced by a fuzzy algorithm, and showed the best recognition rate on a brain MRI dataset.

The authors [10] recently developed a piece of work that used deep learning and transfer learning to classify different MRI pictures of brain tumors. They showed acceptable results on a small public dataset of brain tumor MRI images. Likewise, an artificial neural network (ANN) based approach was developed by [11] that efficiently classified normal and abnormal MRI images. In this approach, they utilized a median filter in the pre-processing step in order to diminish the noise from MRI images. For the feature extraction, they employed the wavelet transform to extract the best features from the enhanced images. Then the dimensions are reduced by employing color moments. Finally, for the classification of normal and abnormal MRI images, the fast-forward ANN has been utilized. They utilized only 70 images for their corresponding experiments. In contrast, a cutting-edge system with three fundamental modules was created by [12] for the identification of brain tumors. They utilized histogram equalization in the pre-processing module in order to enhance the contrast of the brain MRI images. While, in the feature extraction module, they used principal component analysis followed by independent analysis to extract prominent features. Finally, they utilized an integrated classifier that was based on Naïve Bayes recurrent neural networks. They claimed better performance using publicly available brain MRI datasets.

Additionally, [13] established a reliable method for the recognition and segmentation of the brain tumor in MRI images. To distinguish between a wide range of tumor tissues in normal and abnormal MRI images and segment the tumor area accordingly, they used Berkeley’s wavelet transform followed by a deep learning classifier. Most of the afore-mentioned approaches showed better performances and acceptable results on small brain MRI images. However, their performances and classification accuracies are accordingly decreased on large brain MRI datasets.

As a result, this study suggests a precise and effective system for the classification of brain diseases using MRI images. In this approach, the following contributions have been made.

- In the preprocessing step, the MRI images have been enhanced through existing well-known techniques like global histogram equalization.
- Then, for feature extraction, an accurate and robust technique is proposed that is based on symlet wavelet transform. This technique yields better classification outcomes because it can handle the orthogonal, biorthogonal, and reverse biorthogonal features of gray scale images. Our tests support the frequency-based supposition. The wavelet coefficients’ statistical reliance was assessed for each frame of grayscale MRI data. A gray scale frame’s joint probability is calculated by collecting geometrically aligned MRI images for each wavelet coefficient. In order to determine the wavelet coefficients obtained from these distributions, the mutual information between the two MRI images is used to calculate the statistical dependence’s intensity.
- Following the extraction of the best feature, a linear discriminant analysis (LDA) was used to minimize the feature space’s dimensions.
- Following the selection of the best features, the model is trained using logistic regression, which uses the coefficient values to determine which characteristics (i.e., which pixels) are crucial in deciding which class a sample belongs to. The per-class probability for each sample may be computed using the coefficient values, and the conditional probability for each class can be computed using this method. In general, the class with the highest probability might be found to acquire the predicted label.
- In order to assess the performance of the proposed approach, a comprehensive set of experiments was performed using the brain MRI dataset, which has 24 various kinds of brain diseases.

For this assessment, a comprehensive dataset is collected from Harvard Medical School [14] and Open Access Series of Imaging Studies (OASIS) [15], which has total 24 various kinds of diseases such as Fatal stroke (FS), Motor neuron disease (MN), Glioma (GL), Vascular dementia (VD), Cavernous angioma (CA), Hypertensive encephalopathy (HY), Cerebral calcinosis (CC), Metastatic adenocarcinoma (MA), Chronic subdural hematoma (CS), Multiple embolic infarctions (MI), AIDS dementia (AD), Cerebral toxoplasmosis (CT), Meningioma (M), Pick’s disease (PD), Sarcoma (SR), Alzheimer’s disease (AL), Creutzfeld-Jakob disease (CJ), Metastatic bronchogenic carcinoma (MB), Alzheimer’s disease with visual agnosia (AV), Multiple sclerosis (MS), Lyme encephalopathy (LE), Herpes encephalitis (HE), Cerebral haemorrhage (CH), Huntington’s disease (HD), and normal brain (NB). The proposed technique achieved better performance on this comprehensive MRI dataset.

The entire paper is organized as follows: Section 2 describes the existing MRI systems along with their respective disadvantages. Section 3 presents the proposed approach, while, the experimental setup is described in Section 4. Based on the experimental setup, the results are shown in Section 5. Finally, Section 6 summarizes the proposed approach along with future directions.

## 2. Literature Review

In the past couple of years, lots of efficient and accurate studies have been done for the classification of numerous types of brain ailments using MRI images. Most of these studies showed the best performances on a small dataset of brain MRI. However, their performances degraded accordingly on larger testing datasets. Therefore, a robust and accurate framework has been designed that showed good classification results on a large brain MRI dataset.

A novel method has been proposed by [16] that is based on statistical features coupled with various machine learning techniques. They claimed the best performance on a small MRI dataset. However, computational-wise, this approach is much more expensive. A state-of-the-art framework has been designed by [17], which classified the Alzheimer disease using MRI images. In this framework, the corresponding MRI image has been enhanced in the preprocessing step, while the brain tissues are segmented in the post-processing step. Then several deep learning techniques (convolutional neural network) are employed to classify the corresponding disease. However, the convolutional neural network has an overfitting problem [18]. Also, this approach has been tested and validated on a small dataset. Similarly, an accurate and robust method was proposed by [19]. They utilized stepwise linear discriminant analysis (SWLDA) for feature extraction and support vector machines for classification on a large brain MRI dataset. They achieved the best performance using the MRI dataset. However, SWLDA is a linear method that might be employed in a small subspace of binary classification problems [20].

On the other hand, an integrated approach was designed by [21], where the authors integrated a feature-based classifier and an image-based classifier for brain tumor classification. Further, their proposed architecture was based on deep neural networks and deep convolutional networks. They achieved a comparable classification rate. However, a huge number of training images and the carefully constructed deep networks required for this approach [22]. Similarly, a state-of-the-art framework was designed by [23] in order to classify brain MRI along with gender and age. They utilized deep neural network, convolutional network, LeNet, AlexNet, ResNet, and SVM to classify abnormal and normal MRIs accurately. However, they showed better performance on a small dataset, and most of the experiments were in a static environment. Likewise, the authors of [24] proposed an efficient brain image classification system using an MRI dataset. In their system, they extracted the features by shape and textual method, such as region based active contour, and showed good performance. However, the major limitation of the region-based method is its’ sensitivity to the initialization, and because of this, the region of interest does not segment properly [25].

A cutting-edge method for classifying various brain illnesses using MRI images was reported by Nayak et al. [26]. They utilized convolutional neural network-based dense EfficientNet coupled with min-mix normalization for categorization, and they showed better performance using the MRI dataset. However, this approach employs a huge number of operations, which make the model computationally slower [27]. Similarly, an integrated framework was designed by [28], where the authors employed a semantic segmentation network coupled with GoogleNet and a convolutional neural network (CNN) for brain tumor classification using MRI and CT images. They achieved better results using a small dataset of brain MRI and CT. However, in GoogleNet, the connected layers cannot manage various input image sizes [29].

A fully automated brain tumor segmentation approach was developed by [30] that was based on support vector machines and CNN. Moreover, the segmentation was done through the details of various techniques such as structural, morphological, and relaxometry. However, the methodologies utilized in this framework have comparatively lower significance with larger amounts of input MRI images [31]. Because it is a challenging task for these methods to accurately detect the abnormalities in the brain MRI images [31]. Moreover, a modified CNN based model was developed by [32] for the analysis of brain tumors. The authors employed CNN along with parametric optimization techniques such as the sunflower optimization algorithm (SFOA), the forensic-based investigation algorithm (FBIA), and the material generation algorithm (MGA). They claimed the highest accuracy of classification using the MRI dataset. However, SFOA is very sensitive to initializing and premature convergence [33]. Moreover, in MGA, the predictions are made based on single-slice inputs, hypothetically restraining the information available to the network [34].

An integrated framework was proposed by [35], which was based on the VGG19 feature extractor along with a progressive growing generative adversarial network (PGGAN) augmentation model for brain tumor classification using MRI images. They achieved good classification results on a publicly available MRI dataset. However, this approach cannot generate high-resolution images via the PGGAN model [36]. Moreover, this approach might not generate new examples with objects in the desired condition [37]. Another state-of-the-art scheme was proposed by [38], which contained some steps such as preprocessing, segmentation, feature extraction, and classification. The image was enhanced via a Wiener filter followed by edge detection. The tumor was segmented by a mean shift clustering algorithm. The features were extracted from the segmented tumor through the gray level co-occurrence matrix (GLCM), and the classification was done by support vector machines. However, the GLCM method is robust to Gaussian noise, and the extracted features are based on the difference between the corresponding pixels, but the magnitude of the difference was not taken into account [39].

A state-of-the-art fused method was developed by [40] that was based on gray level co-occurrence matrix (GLCM), spatial grey level dependence matrix (SGLDM), and Harris hawks optimization (HHO) techniques followed by support vector machines for brain tumor detection. However, this approach depends on the manual selection of the region of interest, due to which the corresponding results in the dependence of parameter values on the extracted region might not be selected [40].

As a result, in this work, a solid framework was created for the classification of various brain illnesses using an MRI dataset. A symlet wavelet-based feature extraction method was designed and is used in the proposed framework to extract the key features from brain MRI images. Furthermore, the dimensions of the feature space are reduced by LDA, and the classification is done through logistic regression. The proposed approach achieved the best classification results using MRI images compared to the existing publications.

## 3. Proposed Feature Extraction Methodology

**Figure 1:**
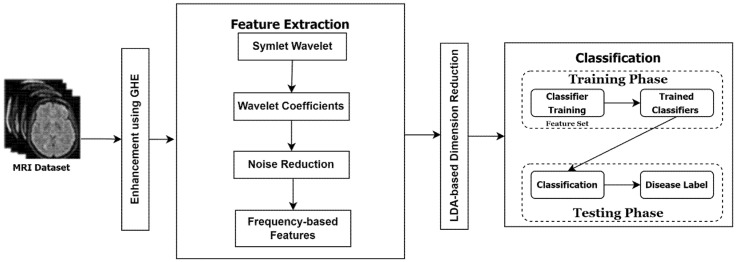
The working diagram of the proposed MRI classification Approach.

The overall working diagram for the proposed brain MRI images is presented in Figure 1.

### 3.1. Preprocessing

**Figure 2:**
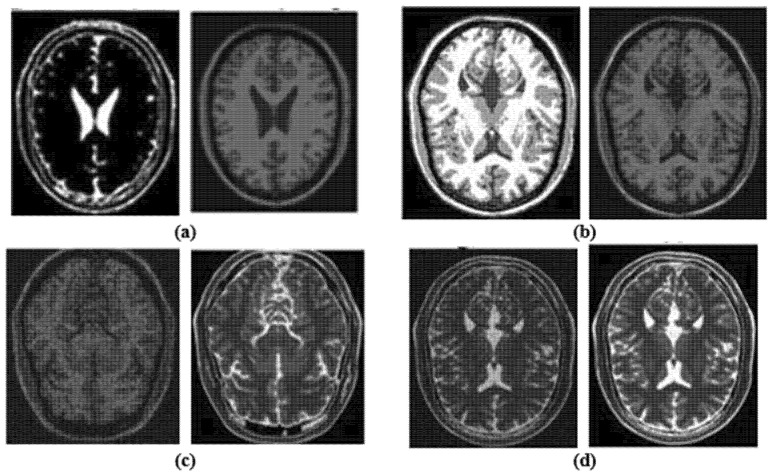
The corresponding results of preprocessing step.

Most images contain extra elements, including background information, lighting effects, and pointless details that could lead to classification errors. To facilitate quick processing and enhance image quality, it is crucial to remove any superfluous parameters. To enhance the quality of the images by extending the dynamic range’s intensity using the histogram of the entire image, the global histogram equalization (GHE) is used in the preprocessing stage. In essence, GHE finds the histogram’s sequential sum, normalizes it, and then multiplies it by the value of the highest gray level. Then, utilizing one-to-one correspondence, these values are translated onto the earlier original values. GHE’s transformation function is given in Equation (1).

(1) $$G_{k}=C\left(g_{k}\right)=\overset{k}{\underset{i=0}{\sum }}P(g_{i})=\overset{k}{\underset{i=0}{\sum }}\frac{n_{i}}{n}$$

where *k =* 0, 1, 2…, *N −* 1, 0 ≤ *G_k_* ≤ 1, *n* is the total number of pixels in the input image,* n_i_* is the number of pixels with the grey level *g_i_*, and *P*(*g_i_*) is the PDF of the input grey level. To evenly distribute the brightness histogram of picture (*I*) in GHE, the image (*I*) must first be normalized before the PDF can be calculated. This is shown by Equation (2),

(2) $$P\left(I_{i}\right)=\frac{n_{i}}{n},\;0\leq I_{i}\leq 1\;\mathrm{and}\;\sum_{i=0}^{N-1}P\left(g_{i}\right)=1$$

where the cumulative density function (CDF) dependent on PDF is denoted by *C(r_k_)* in (1). The supplied transformation function in Equation (1), which is mapped by multiplying it by [*N* − 1], represents the GHE and has a dynamic range of [0, *N* − 1]. This method produced images with a resolution of 240 × 320 pixels. The corresponding results are shown in Figure 2. The Figures (a) and (b) the left side images are affected by light and the right-side images are respectively enhanced by the preprocessing step; while, the Figures (c) and (d), the left side images are affected by noise and the right-side images are respectively enhanced by the preprocessing step.

### 3.2. Symlet Wavelet Transform

Following the preprocessing stage of enhancing the MRI images, the symlet wavelet transform has been used to extract a number of standout features from the MRI images. The decomposition method was employed in this procedure, which required grayscale video frames. The proposed algorithm was converted from RGB to grayscale in order to increase its effectiveness. The decomposition of the signal into a group of distinct feature vectors could be understood as the wavelet decomposition. Each vector includes smaller sub-vectors, such as

(3) $$F_{0}^{2D}=F_{0}^{2D-1},\;F_{0}^{2D-2},\ldots ,F_{0}^{2D-n}$$

where *F* represents the 2D feature vector. Let assume, a 2D MRI image like *Y* that has been divided into orthogonal sub-images for various visualizations. One level of decomposition is depicted in the following equation.

*Y* = *R*_1_ + *P*_1_ (4)

where, *R*_1_ and *P*_1_ denote rough and precise coefficient vectors, respectively, and *Y* denotes the decomposed image. If the MRI image is divided into multiple levels, then, the Equation (3) can be expressed as.

(5) $$Y=R_{j}+\left[P_{j}+P_{j-1}+P_{j-2}+\ldots +P_{2}+P_{1}\right]$$

where, *j* indicates the decomposition’s level. Only the rough coefficients were used for feature extraction because the precise coefficients are typically made up of noise. Each frame is divided into up to four layers of decomposition during the decomposition process, or *j* = 4, because beyond this value, the image loses a lot of information, making it difficult to discover the useful coefficients and perhaps leading to misclassification.

The precise coefficients further consist of three sub-coefficients. So, the Equation (4) can be written as

(6) $$Y=R_{4}+\left[P_{4}+P_{3}+P_{2}+P_{1}\right]\;\;\;\;\;\;\;\;\;\;\;\;\;\;\;\;\;\;\;\;\;\;\;\;\;=R_{4}+\left[{\left(P_{h}\right)}_{4}+{\left(P_{v}\right)}_{4}+{\left(P_{d}\right)}_{4}\right]+\;\;\;\;\;\;\;\;\;\;\;\;\;\;\;\;\;\;\;\;\;\;\;\;\;\;\;\;\;\;\;\;\;\;\;\;\;\;\;\;\left[{\left(P_{h}\right)}_{3}+{\left(P_{v}\right)}_{3}+{\left(P_{d}\right)}_{3}\right]+\;\;\;\;\;\;\;\;\;\;\;\;\;\;\;\;\;\;\;\;\;\;\;\;\;\;\;\;\;\;\;\;\;\;\;\;\;\;\;\;\left[{\left(P_{h}\right)}_{2}+{\left(P_{v}\right)}_{2}+{\left(P_{d}\right)}_{2}\right]+\;\;\;\;\;\;\;\;\;\;\;\;\;\;\;\;\;\;\;\;\;\;\;\;\;\;\;\;\;\;\;\;\;\;\;\;\;\;\;\;\left[{\left(P_{h}\right)}_{1}+{\left(P_{v}\right)}_{1}+{\left(P_{d}\right)}_{1}\right]+$$

**Figure 3:**
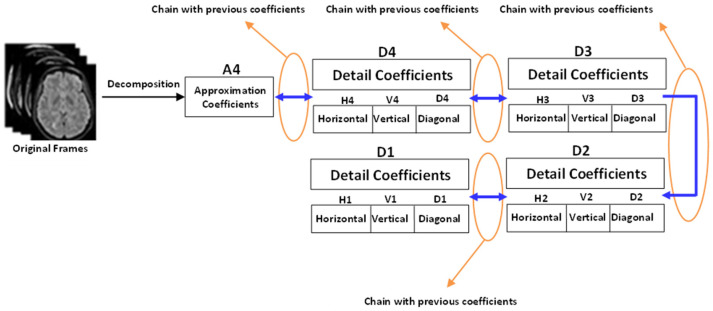
The entire coefficients are connected in the form of a chain.

Or simply, the Equation (5) can be written as

(7) $$Y=R_{4}+\overset{4}{\underset{i=1}{\sum }}\left[{\left(P_{h}\right)}_{i}+{\left(P_{v}\right)}_{i}+{\left(P_{d}\right)}_{i}\right]$$

where, *P_v_, P_h_*, and *P_d_* represent vertical, horizontal, and diagonal coefficients, respectively. As can be seen from Equation (6) or (7), all the coefficients are linked to one another in a chain, making it simple to identify the salient features. Figure 3 graphically displays these coefficients. For each stage of the decomposition, the rough and precise coefficient vectors are produced by passing the signal through low-pass and high-pass filters, respectively.

The feature vector is produced by averaging all the frequencies present in the MRI images following the decomposition procedure. The frequency of each MRI image within a given time window has been calculated by applying the wavelet transform to the analysis of the relevant frame [41].

(8) $$W\left(x,y\right)=\frac{1}{\sqrt{x}}\overset{\infty }{\underset{-\infty }{\int }}y\left(t\right)\varphi_{f,e}\left(\frac{t-y}{x}\right)dt$$

where, $\;\varphi_{f,\;e}$ is the wavelet function for estimating frequency, and *t* is the time. In order to obtain a greater level of judgment for frequency estimation, *x* is the scale of the wavelet between the lower and upper frequency boundaries. Moreover, *y* represents the wavelet’s position within the time frame with respect to the signal sampling period, and the wavelet coefficients with the supplied scale and position parameters are denoted by *W*(*a_i_*, *b_j_*), and their mode frequency conversion is shown below.

(9) $$f_{1}=\frac{f_{a}\left(\varphi_{f,e}\right)}{a_{m}\left(\varphi_{f,e}\right)}$$

where $f_{a}\left(\;\varphi_{f,\;e}\right)$ is the wavelet function’s average frequency, and δ is the sampling period of the signal. In order to obtain the feature vector, the entire image frequencies for each MRI are averaged as follows:

(10) $$f_{avg}=\frac{f_{1}+f_{2}+f_{3}+\ldots ,+f_{K}}{K}$$

where, *K* denotes the total number of frames for every MRI image, $f_{K}$ is the last frame of the current disease, and *f_avg_* denotes the average values of the frequency for every MRI image. It is also a feature vector for that MRI.

### 3.3. Feature Selection and Dimension Reduction via Linear Discriminant Analysis (LDA)

LDA ensures maximum separability by maximizing the ratio of between-class variation to within-class variance in any given data set. LDA is used to classify data in order to solve speech recognition classification issues. The input is mapped into the classification space, where the samples’ class identification is determined by an ideal linear discriminant function produced by LDA. When the within-class frequencies are unequal and their performances have been evaluated using test data generated at random, LDA handles the situation with ease. The following equations are used to compare within-class *VAR_W_* and between-class *VAR_B_*.

(11) $$VAR_{B}=\overset{c}{\underset{i=1}{\sum }}V_{i}\left(\bar{m_{i}}-\overset{̿}{m}\right){\left(\bar{m_{i}}-\overset{̿}{m}\right)}^{T}$$

(12) $$VAR_{B}=\overset{c}{\underset{i=1}{\sum }}\underset{m_{k}\epsilon C_{i}}{\sum }\left(m_{k}-\bar{m_{i}}\right){\left(m_{k}-\bar{m_{i}}\right)}^{T}$$

where, c is the total number of classes (in our case, *c* represents the total MRI diseases within each state), and *V_i_* represents the vector in the *i*th class *C_i_*. Also, $\bar{m}$ represents the mean of the class *C_i_*, *m_k_* represents the vector of a specific class, and $\overset{̿}{m}\;$ represents the mean of all vectors. The optimal projection matrix for discrimination, *D_o_*, is taken by maximizing the determinant of the between-class and within-class scatter matrices, and it is selected as

(13) $$D_{0}=\begin{array}{c} argmax\\ D \end{array}\frac{\left|D^{T}VAR_{B}D\right|}{\left|D^{T}VAR_{W}D\right|}={\left[d_{1},\;d_{2},\ldots ,\;d_{t}\right]}^{T}$$

where, *D_o_* is the collection of discriminate vectors of *VAR_W_* and *VAR_B_* that correspond to the *c −* 1 highest generalized Eigen values ω. *D_o_* has a size of t×r (t≤r), and *r* is the dimension of a vector. Then,

(14) $$VAR_{B}d_{i}=\omega_{i}VAR_{W}d_{i},\;i=1,\;2,\ldots ,\;c-1$$

where, the upper bound value of t is *c −* 1, and the rank of *VAR_B_* is *c −* 1 or less.

Thus, LDA minimizes the within scatter of classes like MRI diseases while maximizing the total dispersion of the data. Please refer to [42] for additional information on LDA.

### 3.4. Classification via Logistic Regression

A popular linear model that can be used for image categorization is logistic regression. In this model, a logistic function is used to simulate the probabilities describing the potential outcomes of a single experiment.

The example of logistic regression can be binary, e.g., One-vs-Rest, or multinomial logistic regression with optional regularization of *ℓ*_1_, *ℓ*_2_ or Elastic-Net.

As an optimization problem, binary class *ℓ*_2_ regularized logistic regression optimizes the following cost function:

(15) $$\underset{w,c}{\min}\frac{1}{2}w^{T}w+C\overset{n}{\underset{i=1}{\sum }}log\left(exp\left(-y_{i}\left(X_{i}^{T}w+c\right)\right)\right)$$

Similarly, ℓ_1_ regularized logistic regression optimizes the following cost function:

(16) $$\underset{w,c}{\min}ǁw{ǁ}_{1}+C\overset{n}{\underset{i=1}{\sum }}log\left(exp\left(-y_{i}\left(X_{i}^{T}w+c\right)\right)\right)$$

Elastic-Net regularization is a combination of ℓ_1_ and ℓ_2_, and minimizes the following cost function:

(17) $$\underset{w,c}{\min}\frac{1-\partial }{2}\;w^{T}w+\partial ǁw{ǁ}_{1}+C\overset{n}{\underset{i=1}{\sum }}\log\left(\exp\left(-y_{i}\left(X_{i}^{T}w+c\right)\right)+1\right)$$

where, ∂ regulates the relative magnitude of *ℓ*_1_ regularization vs. *ℓ*_2_ regularization. Note that, in this notation, the target *y_i_* is supposed to accept values from the set [−1, 1] at trial *i*. Additionally, The Elastic-Net is identical, as might be demonstrated to *ℓ*_1_ when *ρ* = 1 and to *ℓ*_2_ when *ρ* = 0. Please see [43] for a comprehensive detail of logistic regression.

## 4. Designed Approach Evaluation

The proposed technique is evaluated in the following order to show the performance of the proposed technique.

### 4.1. MRI Images Dataset

A comprehensive and generalized MRI dataset was created that contained the actual MRI images from the Harvard Medical School and OASIS MRI databases. The collection contains brain MRI images that have been T1 and T2 weighted. Each input image is 256 × 256 × 3 pixels in size and contains demographic and clinical data, including the patients’ gender, age, clinical dementia rating, mental state observation, and test parameters. The patients are all right-handed. This dataset is separated into two groups: the first comprises eleven diseases (which is used as a benchmark dataset by most existing works), and the second contains 24 diseases, including eleven from the first group. For large-scale experiments, this group is more ubiquitous. The overall number of brain MRI images in the first group is 255 (220 abnormal and 35 normal), while the total number of images in the second group is 340 (260 abnormal and 80 normal).

### 4.2. Experiment Settings

The performance of the created approach is assessed using the extensive set of experiments below, which are carried out in MATLAB using the specifications of RAM 8GB and processor running at 1.7 Hz.

- The first experiment is implemented in order to assess the significance of the developed method on a publicly available MRI dataset. The entire experiment is performed against an *n*-fold cross validation scheme, where every image is used for both training and testing.
- While, the second experiment presents the importance of the proposed technique in the MRI classification system. For which, a comprehensive sub experiments were executed; where, well-known existing feature extraction algorithms were employed like Speeded Up Robust Features (SURF), Gray Texture Features, Fusion Feature, Least Squares, Partial, Semidefinite Embedding, Latent Semantic Analysis, Independent Component Analysis (ICA) instead of the developed approach.
- Finally, the third experiment prescribes the comparison of the developed approach against the state-of-the-art systems. This experiment was performed against three major measurement rules such as sensitivity, accuracy, and specificity, which are measured through the values of false positive and false negative.

## 5. Experimental Results

The performance of the proposed approach is evaluated through the following comprehensive set of experiments, which are presented in the following order.

### 5.1. 1st Experiment

This experiment presents the significance of the developed technique on the brain MRI dataset. An *n*-fold cross validation rule was used, where every MRI image has been used accordingly for training and validation. Table 1 contains the performance of the proposed approach.

Table 1 clarifies that the developed technique achieved the best classification rates on a large brain MRI dataset. This is because the statistical reliance of the wavelet coefficients is measured in the proposed method, which means that the joint probabilities are calculated by collecting geometrically aligned MRI images for each wavelet coefficient. In order to determine the wavelet coefficients obtained from these distributions, the mutual information between the two MRI images is used to calculate the statistical dependence’s intensity. The execution time for the classification of every class using the proposed approach was 21.5 s against brain MRI dataset, which shows that the proposed approach was not only accurate but also computational wise less expensive.

### 5.2. 2nd Experiment

In the second type of experiment, a number of tests were performed to demonstrate the value of the suggested feature extraction method for the classification of brain MRI images. The existing state-of-the-art feature extraction techniques are employed instead of utilizing the proposed feature extraction method in the MRI system. The same experimental setup is kept for these experiments as in the first experiment. Then Speeded Up Robust Features, Gray Texture Features, Fusion Feature, Latent Semantic Analysis, Partial, Least Squares, Semidefinite Embedding, Independent Component Analysis are employed in the current respective MRI system. The entire results are presented in Table 2, Table 3, Table 4, Table 5, Table 6,Table 7 and Table 8.

It is evident from Table 2, Table 3, Table 4, Table 5, Table 6,Table 7 and Table 8 that the corresponding MRI classification system did not achieve better recognition rates using the existing well-known feature extraction methods. Hence, the importance of the proposed feature extraction method might be judged by the respective MRI disease classification system. This is because the proposed approach can handle the orthogonal, biorthogonal, and reverse biorthogonal properties of gray scale images, and produces higher classification results. Our experiments validate the frequency-based assumption. The statistical reliance on wavelet coefficients is assessed for all grayscale MRI images. A gray scale frame’s joint probability is calculated by collecting geometrically aligned MRI pictures for each wavelet coefficient. In order to determine the wavelet coefficients obtained from these distributions, the mutual information between the two MRI images is used to calculate the statistical dependence’s intensity.

### 5.3. 3rd Experiment

Finally, in this experiment, we have compared the recognition rate of the proposed approach against existing state-of-the-art systems. These systems were implemented using the existing settings as described in their respective articles. For some systems, we have borrowed their respective implementation, while for others we have utilized their results as mentioned in their respective studies for a fair comparison. Moreover, the proposed approach and the existing state-of-the-art methods are measured through different measurement schemes such as sensitivity, accuracy, and specificity. For every measurement, we utilized the following formulas for evaluation.

(18) $$Sensitivity=\frac{T_{p}}{T_{p}+F_{p}}$$

(19) $$Accuracy=\frac{T_{p}+T_{n}}{T_{p}+F_{p}+T_{n}+F_{n}}$$

(20) $$Specificity=\frac{T_{n}}{T_{n}+F_{p}}$$

where $T_{p}$ is true positive, $T_{n}$ is true negative, $F_{p}$ is false positive, and $F_{n}$ is false negative. The entire comparisons against the afore-mentioned measurements are respectively presented in Table 9, Table 10 and Table 11.

Table 9 denotes that the designed framework achieved remarkable achievements compared to the state-of-the-art studies. This is the proposed frameworks that handles the orthogonal, biorthogonal, and reverse biorthogonal properties of gray scale images. It produces higher classification results.

Similarly, Table 10 presents the effectiveness of the proposed approach. A comparison has been made against state-of-the-art methods in terms of true positive, true negative, false positive, and false negative.

Likewise, Table 11 provides a comparison between the proposed approach and the existing studies in terms of accuracy, sensitivity, and specificity. As can be seen, the proposed approach provides better sensitivity and specificity results compared with existing state-of-the-art methods.

## 6. Conclusions

In medical imaging, magnetic resonance imaging (MRI) is a precise and noninvasive technique that can be used to diagnose a variety of disorders. Various algorithms for brain MRI categorization have been developed by a number of researchers. On small MRI datasets, the majority of these algorithms did well and had higher identification rates. When dealing with larger MRI datasets, however, their performance degrades. As a result, the objective is to create a quick and precise classification system that can sustain a high identification rate across a sizable MRI dataset. As a result, in this study, a well-known enhancement method called global histogram equalization (GHE) is used to reduce undesirable information in MRI images. Furthermore, a reliable and accurate feature extraction technique is suggested for extracting and selecting the most prominent feature from an MRI picture. The suggested feature extraction method for grayscale photos is a compactly supported wavelet that has the greatest number of vanishing moments and the least amount of asymmetry for a given support width. Our study supports the frequency-based hypothesis. The statistical dependence of the wavelet coefficients is assessed for all grayscale MRI pictures. A gray scale frame’s joint probability is calculated by collecting geometrically aligned MRI pictures for each wavelet coefficient. Using mutual information for the wavelet coefficients derived using these distributions, the degree of statistical dependence between the two MRI images is evaluated. Furthermore, the linear discriminant analysis is used after extracting the features to choose the best features and lower the dimensions of the feature space, which may improve the performance of the recommended method for generating feature vectors. Finally, logistic regression is used to classify the brain illnesses. A huge dataset from Harvard Medical School and the OASIS is utilized, which comprises a total of 24 distinct types of brain disorders, to assess and test the suggested method.

In the proposed approach, the optimum set of features is extracted from the MRI images that are important for improving the accuracy. Subsequently, the rate of convergence is also one of the main factors improving the accuracy of this research; however, the number of features in this approach is not too high to reduce the computational complexity. Therefore, in the future, the proposed approach will be enclosed using MRI datasets in various healthcare domains. Moreover, the proposed approach is robust and efficient, which might be useful for real-time diagnostic applications in the future. Therefore, the proposed method might play a significant role in helping the radiologists and physicians with the initial diagnosis of the brain diseases using MRI.

## Figures and Tables

**Table 1:** Performance of the developed approach using MRI dataset (Unit %).

**Illnesses**	**FS**	**MN**	**GL**	**VD**	**CA**	**HY**	**CC**	**MA**	**CS**	**MI**	**AD**	**CT**	**ME**	**PD**	**SR**	**AL**	**CJ**	**MB**	**AV**	**MS**	**LE**	**HE**	**CH**	**HD**	**NB**
**FS**	**96**	0	0	0	1	0	0	0	0	2	0	0	0	0	0	0	0	0	0	1	0	0	0	0	0
**MN**	0	**98**	0	0	0	0	0	0	0	0	0	0	1	0	0	0	0	1	0	0	0	0	0	0	0
**GL**	1	0	**99**	0	0	0	0	0	0	0	0	0	0	0	0	0	0	0	0	0	0	0	0	0	0
**VD**	0	0	0	**100**	0	0	0	0	0	0	0	0	0	0	0	0	0	0	0	0	0	0	0	0	0
**CA**	0	0	1	0	**95**	0	0	2	0	0	0	0	0	0	0	1	0	0	0	0	0	0	0	0	1
**HY**	2	0	0	0	0	**93**	0	0	0	0	0	2	0	0	1	0	0	0	0	0	0	2	0	0	0
**CC**	0	0	0	0	0	1	**99**	0	0	0	0	0	0	0	0	0	0	0	0	0	0	0	0	0	0
**MA**	0	0	0	0	2	0	0	**97**	0	0	1	0	0	0	0	0	0	0	0	0	0	0	0	0	0
**CS**	0	1	0	0	0	0	1	0	**96**	0	0	0	0	2	0	0	0	0	0	0	0	0	0	0	0
**MI**	1	0	0	1	0	0	0	0	2	**94**	0	0	0	0	0	0	1	0	0	0	1	0	0	0	0
**AD**	0	0	0	0	0	0	0	0	0	1	**97**	0	0	0	0	2	0	0	0	0	0	0	0	0	0
**CT**	0	0	1	0	0	0	0	0	0	0	0	**98**	1	0	0	0	0	0	0	0	0	0	0	0	0
**ME**	0	0	0	0	0	0	0	0	0	0	0	0	**100**	0	0	0	0	0	0	0	0	0	0	0	0
**PD**	2	0	0	1	0	0	0	1	0	0	0	0	0	**94**	0	0	0	0	0	0	0	0	0	0	2
**SR**	0	0	0	0	1	0	0	0	0	0	2	0	0	0	**96**	1	0	0	0	0	0	0	0	0	0
**AL**	0	1	0	0	0	0	0	0	0	0	0	0	0	0	0	**97**	0	0	2	0	0	0	0	0	0
**CJ**	0	0	0	0	0	0	0	0	0	0	0	1	0	0	0	0	**99**	0	0	0	0	0	0	0	0
**MB**	1	0	0	0	0	2	0	0	0	0	1	0	0	0	0	0	0	**94**	0	0	0	0	2	0	0
**AV**	0	0	0	0	0	0	0	1	0	0	0	0	2	0	0	0	0	1	**95**	0	0	0	0	1	0
**MS**	0	0	1	0	0	0	0	0	0	1	0	0	0	0	2	0	0	0	0	**96**	0	0	0	0	0
**LE**	0	0	0	0	2	0	0	0	0	0	0	0	0	0	0	0	0	0	0	0	**98**	0	0	0	0
**HE**	0	0	0	0	0	0	0	0	0	0	0	0	0	1	0	0	0	0	0	0	0	**99**	0	0	0
**CH**	0	1	0	0	0	0	2	0	0	0	0	0	0	0	0	0	1	0	0	0	0	2	**94**	0	0
**HD**	2	0	0	1	0	0	0	0	0	0	0	2	0	0	0	0	0	0	0	1	0	0	0	**93**	1
**NB**	0	0	0	0	0	1	0	0	0	0	0	0	0	0	0	0	0	2	0	0	0	0	0	0	**97**
**Average**	**96.58%**																								

**Table 2:** Performance of Speeded Up Robust Features (SURF) method (instead of using the developed approach) using MRI dataset (Unit %).

**Illnesses**	**FS**	**MN**	**GL**	**VD**	**CA**	**HY**	**CC**	**MA**	**CS**	**MI**	**AD**	**CT**	**ME**	**PD**	**SR**	**AL**	**CJ**	**MB**	**AV**	**MS**	**LE**	**HE**	**CH**	**HD**	**NB**
**FS**	**87**	0	2	0	0	0	1	0	2	0	0	2	0	0	0	2	0	0	1	0	1	0	0	2	0
**MN**	2	**88**	0	0	1	0	0	0	0	2	0	0	1	0	1	0	0	0	0	2	0	0	1	0	2
**GL**	0	1	**90**	0	0	2	0	1	0	0	0	1	0	0	0	0	2	0	0	0	1	0	2	0	0
**VD**	1	0	0	**85**	2	0	1	0	1	0	2	0	0	3	0	0	0	2	0	1	0	0	0	2	0
**CA**	2	0	3	1	**80**	0	0	2	0	1	1	0	2	0	2	1	0	0	2	0	1	2	0	0	0
**HY**	0	1	0	0	0	**85**	2	0	1	0	0	2	0	1	0	0	2	0	0	2	0	0	2	0	2
**CC**	0	0	1	0	2	0	**88**	1	0	0	2	0	2	0	1	0	0	1	0	0	0	2	0	0	0
**MA**	2	0	0	2	0	2	0	**82**	0	2	0	1	0	2	0	2	1	0	2	0	1	0	0	1	0
**CS**	0	2	1	0	0	0	4	0	**83**	0	2	0	1	0	2	0	0	1	0	2	0	0	1	0	1
**MI**	1	0	0	2	1	0	1	0	0	**85**	0	2	0	2	0	0	1	0	2	0	0	1	0	2	0
**AD**	0	1	0	0	0	2	0	2	0	1	**87**	0	1	0	0	2	0	1	0	0	2	0	0	0	1
**CT**	0	0	2	0	0	0	2	0	1	0	2	**91**	0	0	1	0	0	0	0	1	0	0	0	0	0
**ME**	2	1	0	1	2	0	0	1	0	2	0	1	**79**	3	0	2	1	0	2	0	1	0	2	0	0
**PD**	0	1	2	0	0	1	2	0	2	0	1	0	2	**80**	2	0	1	2	0	1	0	2	0	1	0
**SR**	1	0	0	2	1	0	0	1	0	2	0	2	0	0	**81**	2	0	1	2	0	1	0	2	0	2
**AL**	2	0	1	0	0	2	0	0	2	0	1	0	1	2	0	**82**	2	0	0	2	0	1	0	2	0
**CJ**	0	1	0	2	0	0	1	2	0	1	0	2	0	0	1	0	**86**	1	0	0	2	0	1	0	0
**MB**	0	0	2	0	1	2	0	0	0	0	2	0	0	1	0	1	0	**88**	1	0	0	0	0	0	2
**AV**	1	0	0	2	0	0	1	0	2	0	0	1	0	0	0	0	0	0	**90**	2	0	1	0	0	0
**MS**	0	2	0	0	0	1	0	2	0	1	0	0	2	0	1	0	1	0	0	**89**	0	0	0	1	0
**LE**	2	0	1	0	2	0	0	0	1	0	2	0	0	1	0	0	0	2	1	0	**84**	0	2	0	2
**HE**	0	1	0	2	0	0	2	0	0	2	0	1	0	0	1	2	0	0	0	2	0	**86**	0	1	0
**CH**	1	0	0	0	1	0	0	1	0	0	1	0	2	0	0	0	2	0	2	0	1	0	**87**	0	2
**HD**	0	0	2	0	0	2	1	0	2	0	0	1	0	1	2	0	0	1	0	1	0	2	3	**82**	0
**NB**	2	1	0	1	0	0	2	2	0	1	1	0	2	0	0	2	1	0	1	0	2	0	0	1	**81**
**Average**	**85.04%**																								

**Table 3:** Performance of Gray Texture Features method (instead of using the developed approach) using MRI dataset (Unit %).

**Illnesses**	**FS**	**MN**	**GL**	**VD**	**CA**	**HY**	**CC**	**MA**	**CS**	**MI**	**AD**	**CT**	**ME**	**PD**	**SR**	**AL**	**CJ**	**MB**	**AV**	**MS**	**LE**	**HE**	**CH**	**HD**	**NB**
**FS**	**72**	0	4	2	1	2	0	0	2	0	1	2	2	0	0	1	2	1	2	0	2	2	0	0	2
**MN**	1	**69**	0	2	0	1	4	2	0	2	2	0	4	2	1	0	1	0	2	2	1	0	2	2	0
**GL**	0	1	**78**	0	2	2	1	0	1	0	0	1	0	1	2	2	0	2	1	0	2	2	0	1	1
**VD**	2	0	1	**76**	0	0	3	0	1	3	0	0	1	2	0	1	1	3	4	0	1	0	1	0	0
**CA**	0	3	1	0	**77**	2	0	0	4	0	1	2	0	0	2	1	0	0	3	1	0	1	1	0	1
**HY**	3	1	0	2	0	**71**	2	2	0	2	0	1	1	1	2	0	0	3	4	0	1	2	0	1	1
**CC**	0	2	1	1	0	0	**68**	3	2	0	1	1	2	1	4	3	0	2	0	4	2	1	2	0	0
**MA**	1	3	0	0	1	2	2	**70**	3	2	1	3	2	1	0	0	2	1	1	2	0	1	1	0	1
**CS**	0	0	2	0	1	1	0	0	**80**	1	1	0	0	1	0	0	1	2	3	0	1	2	2	1	1
**MI**	1	0	0	0	0	2	1	2	1	**81**	0	3	2	0	1	0	1	1	2	0	0	1	1	0	0
**AD**	1	1	1	1	2	1	0	0	3	1	**74**	1	1	2	1	1	0	0	3	2	0	2	1	0	1
**CT**	1	0	0	3	2	0	2	1	0	1	1	**73**	2	1	1	4	0	2	1	0	2	1	0	2	0
**ME**	0	2	2	0	1	0	1	2	1	0	2	1	**69**	4	0	0	3	1	3	1	1	0	3	2	1
**PD**	2	0	0	1	1	3	1	1	0	3	2	1	1	**66**	2	4	1	2	2	0	3	1	2	0	1
**SR**	1	3	0	0	2	1	1	1	4	1	2	1	3	1	**72**	0	0	0	1	0	0	2	2	0	2
**AL**	0	1	0	1	0	2	2	1	0	0	1	2	3	1	1	**73**	1	2	3	1	0	2	0	1	2
**CJ**	1	2	2	0	1	1	2	1	1	1	2	0	0	1	2	2	**75**	0	1	0	2	0	1	1	1
**MB**	0	0	1	0	2	0	0	3	0	1	0	2	1	0	1	0	1	**79**	3	0	1	1	0	1	3
**AV**	2	1	1	2	1	3	3	0	0	0	1	1	0	3	1	1	2	0	**68**	2	1	2	1	2	2
**MS**	3	1	2	0	0	2	0	1	1	0	2	0	3	2	1	0	0	2	5	**69**	0	1	2	1	2
**LE**	0	1	1	2	3	4	1	2	1	3	0	0	1	0	2	1	1	0	2	3	**67**	0	2	3	0
**HE**	0	3	2	0	0	2	0	2	2	4	0	0	0	0	2	3	1	1	1	2	2	**70**	2	0	1
**CH**	1	2	0	3	1	0	1	0	1	0	2	2	2	3	1	0	0	1	2	3	1	0	**71**	1	2
**HD**	2	0	3	1	2	1	0	3	0	1	1	2	1	0	0	0	0	2	1	1	0	0	2	**74**	3
**NB**	1	2	1	1	0	2	0	1	1	0	2	1	0	0	0	1	2	3	1	0	2	1	0	2	**76**
**Average**	**72.72%**																								

**Table 4:** Performance of Fusion Feature method (instead of using the developed approach) using MRI dataset (Unit %).

**Illnesses**	**FS**	**MN**	**GL**	**VD**	**CA**	**HY**	**CC**	**MA**	**CS**	**MI**	**AD**	**CT**	**ME**	**PD**	**SR**	**AL**	**CJ**	**MB**	**AV**	**MS**	**LE**	**HE**	**CH**	**HD**	**NB**
**FS**	**70**	2	3	1	0	1	2	2	1	0	1	1	0	2	1	2	0	0	0	2	4	2	1	0	2
**MN**	2	**66**	0	1	3	4	1	1	0	3	2	1	3	1	1	1	0	3	0	1	3	1	0	1	1
**GL**	0	1	**67**	1	2	0	2	1	1	2	0	0	2	2	3	2	2	1	3	0	1	2	3	0	2
**VD**	1	0	1	**71**	1	2	0	3	0	3	2	2	1	0	2	0	1	2	1	1	2	0	2	1	1
**CA**	0	0	1	1	**72**	2	1	2	1	1	1	0	2	3	0	2	3	0	0	0	2	2	1	3	0
**HY**	1	1	0	0	2	**73**	2	1	1	2	0	1	1	2	1	0	2	1	2	1	1	2	3	0	0
**CC**	3	0	1	1	0	1	**74**	2	1	3	1	0	0	2	1	1	0	2	0	3	0	1	0	2	1
**MA**	0	1	0	0	1	1	1	**77**	2	0	0	2	2	1	1	2	0	0	0	2	1	2	2	0	2
**CS**	1	2	2	1	2	0	2	2	**66**	1	2	1	1	2	0	0	2	0	2	1	1	2	2	1	4
**MI**	3	1	0	0	0	4	0	0	0	**64**	5	0	0	1	1	1	0	4	5	0	4	0	3	2	2
**AD**	1	2	1	1	2	1	1	2	2	1	**68**	2	3	1	0	0	1	2	1	1	0	1	2	1	3
**CT**	0	0	2	0	2	1	0	0	0	0	2	**79**	1	2	1	1	0	0	2	0	2	3	1	0	1
**ME**	2	1	0	2	3	0	3	2	4	0	0	5	**60**	2	2	3	2	1	1	1	0	4	1	0	1
**PD**	4	0	2	0	0	3	1	1	2	1	0	0	2	**71**	3	2	0	1	0	3	1	2	1	0	0
**SR**	1	1	0	1	1	0	0	3	1	0	0	2	1	1	**73**	1	0	1	2	1	3	0	2	3	2
**AL**	1	2	1	2	0	2	1	0	0	0	1	1	1	1	2	**76**	0	1	2	0	0	2	2	1	1
**CJ**	3	1	0	0	1	0	1	2	1	1	1	0	0	0	3	1	**77**	2	2	1	0	0	2	0	1
**MB**	2	3	1	4	3	1	0	1	3	0	6	0	1	0	1	0	0	**67**	1	1	0	3	0	1	1
**AV**	0	2	1	3	1	0	1	2	0	3	1	1	2	2	2	1	1	2	**69**	1	0	2	1	2	0
**MS**	1	3	2	0	1	1	0	0	2	4	1	1	3	0	1	0	0	2	1	**72**	2	1	0	1	1
**LE**	1	0	1	1	1	2	1	1	3	0	2	0	0	0	0	1	2	2	1	1	**75**	1	1	1	2
**HE**	2	1	1	0	0	1	3	0	0	0	2	3	1	1	1	0	0	2	1	1	2	**74**	1	0	3
**CH**	3	0	0	2	3	4	1	1	3	2	1	0	0	0	3	2	2	1	3	0	1	0	**66**	2	0
**HD**	0	3	2	0	1	1	1	0	3	5	0	2	1	1	2	3	1	2	0	1	1	2	1	**65**	2
**NB**	2	1	2	3	0	0	0	2	1	4	3	2	1	1	0	0	4	2	2	0	0	1	3	2	**64**
**Average**	**70.24%**																								

**Table 5:** Performance of Latent Semantic Analysis method (instead of using the developed approach) using MRI dataset (Unit %).

**Illnesses**	**FS**	**MN**	**GL**	**VD**	**CA**	**HY**	**CC**	**MA**	**CS**	**MI**	**AD**	**CT**	**ME**	**PD**	**SR**	**AL**	**CJ**	**MB**	**AV**	**MS**	**LE**	**HE**	**CH**	**HD**	**NB**
**FS**	**65**	0	2	2	0	1	2	4	0	2	1	2	1	4	0	2	2	2	1	0	2	2	2	0	1
**MN**	2	**66**	1	0	2	2	0	1	2	2	0	1	2	2	2	0	4	1	2	2	2	0	1	3	0
**GL**	1	2	**71**	2	1	0	2	2	1	0	2	1	1	4	0	2	1	0	1	0	2	2	0	0	2
**VD**	0	1	2	**69**	2	2	1	0	2	1	0	4	2	1	2	0	2	2	2	1	1	0	2	1	0
**CA**	2	2	3	1	**67**	0	0	2	1	3	2	1	0	0	1	3	1	1	2	1	0	2	1	2	2
**HY**	3	1	2	1	1	**64**	2	1	1	2	4	0	1	2	0	0	0	2	1	2	1	2	3	1	3
**CC**	4	0	1	3	2	4	**60**	0	2	0	2	3	2	2	1	2	1	3	2	0	2	0	1	2	1
**MA**	0	2	2	1	0	2	4	**61**	1	3	2	0	1	2	2	3	2	0	2	2	1	2	2	0	4
**CS**	2	1	0	2	2	1	0	2	**67**	2	0	1	2	1	2	0	1	2	2	2	3	0	1	2	2
**MI**	1	2	2	1	2	0	2	1	2	**69**	2	0	1	2	0	2	2	0	1	2	1	2	0	1	2
**AD**	2	1	1	0	1	2	0	2	0	2	**72**	1	0	2	2	1	0	2	2	0	2	2	2	1	0
**CT**	0	2	2	1	2	0	2	1	2	0	1	**73**	2	0	1	2	2	1	2	1	0	1	0	2	0
**ME**	1	0	1	2	0	2	1	0	1	2	2	2	**75**	1	2	0	1	2	0	2	1	0	1	0	1
**PD**	0	1	0	1	2	1	0	2	0	1	0	1	0	**77**	1	2	2	0	1	1	2	1	0	2	2
**SR**	2	1	2	0	1	0	2	0	1	0	2	0	1	0	**78**	1	0	1	2	2	0	2	1	1	0
**AL**	1	2	1	2	0	2	1	2	2	2	0	1	0	2	1	**71**	2	2	0	1	2	1	0	2	1
**CJ**	2	0	2	1	2	4	0	1	2	1	2	2	2	1	3	0	**63**	1	2	2	1	2	2	0	2
**MB**	3	2	2	4	1	0	2	2	1	1	3	0	1	2	0	1	1	**61**	2	3	1	0	2	3	2
**AV**	2	3	2	0	1	2	3	0	2	0	2	1	4	0	3	2	0	5	**60**	0	2	2	1	3	0
**MS**	0	2	2	1	1	2	5	3	0	2	2	2	1	3	4	0	3	2	0	**59**	2	1	2	0	1
**LE**	1	1	1	0	2	1	2	0	3	1	0	0	2	1	0	2	1	2	4	0	**71**	2	0	1	2
**HE**	3	2	0	0	0	1	2	2	3	1	1	0	1	1	0	0	0	2	1	3	1	**73**	2	1	0
**CH**	0	1	3	1	1	2	0	1	0	1	0	1	0	2	2	2	1	2	3	1	0	0	**74**	1	1
**HD**	2	2	0	0	3	1	1	1	0	0	2	0	1	0	0	0	2	4	0	0	1	1	1	**76**	2
**NB**	1	1	1	2	2	0	0	0	1	1	2	0	0	1	1	0	0	0	1	1	2	2	1	2	**78**
**Average**	**68.80%**																								

**Table 6:** Performance of Partial Least Squares method (instead of using the developed approach) using MRI dataset (Unit %).

**Illnesses**	**FS**	**MN**	**GL**	**VD**	**CA**	**HY**	**CC**	**MA**	**CS**	**MI**	**AD**	**CT**	**ME**	**PD**	**SR**	**AL**	**CJ**	**MB**	**AV**	**MS**	**LE**	**HE**	**CH**	**HD**	**NB**
**FS**	**61**	0	2	1	2	0	4	2	1	0	4	2	1	2	3	0	2	2	1	0	2	4	1	0	3
**MN**	2	**67**	1	2	0	2	1	3	2	1	0	1	2	4	0	2	1	2	2	2	0	1	0	2	0
**GL**	1	2	**70**	0	2	1	0	2	1	0	2	3	0	1	2	1	2	0	1	2	2	0	2	1	2
**VD**	0	1	2	**74**	1	2	2	0	1	1	2	0	2	0	1	2	1	2	0	1	1	2	1	0	1
**CA**	2	2	0	1	**73**	1	0	2	0	2	1	2	1	1	2	0	2	1	2	2	0	1	0	2	0
**HY**	1	0	2	2	2	**63**	1	2	1	2	4	2	0	3	1	2	1	2	2	0	2	0	2	1	2
**CC**	2	2	1	0	3	2	**62**	1	2	0	1	4	2	1	2	1	2	0	1	2	1	2	4	0	2
**MA**	0	1	2	2	1	2	2	**60**	1	2	0	1	2	2	3	4	0	2	3	1	2	1	0	4	2
**CS**	4	2	1	2	2	1	0	2	**59**	1	2	2	1	2	1	2	2	3	0	2	4	2	1	2	0
**MI**	1	2	2	1	0	2	2	1	2	**66**	1	2	4	1	0	1	2	1	2	1	0	1	2	2	1
**AD**	2	0	1	2	2	2	1	2	0	2	**68**	1	2	2	1	2	1	0	1	2	1	2	1	0	2
**CT**	1	2	2	0	1	0	2	1	2	0	2	**71**	0	2	2	1	2	1	2	0	2	1	0	2	1
**ME**	2	1	0	2	2	1	2	0	1	2	2	0	**68**	1	2	2	0	2	1	2	2	0	2	1	2
**PD**	0	2	2	1	2	2	1	2	4	1	0	3	2	**61**	1	2	2	1	2	1	0	2	1	2	3
**SR**	2	2	1	2	1	2	2	1	0	2	2	1	2	2	**60**	0	1	4	1	2	2	3	2	1	2
**AL**	1	0	2	1	2	1	2	0	2	1	2	2	1	2	2	**64**	2	0	2	1	2	2	1	4	1
**CJ**	2	2	1	2	1	2	0	2	1	2	1	1	2	0	2	2	**66**	2	1	2	1	1	2	0	2
**MB**	1	2	0	1	0	1	2	1	2	0	2	2	1	2	1	2	2	**69**	2	0	2	2	1	2	0
**AV**	2	1	2	0	2	0	1	0	1	2	2	0	2	1	2	0	2	2	**72**	2	1	0	2	0	1
**MS**	0	2	1	2	1	2	0	2	2	1	0	2	1	1	0	1	1	0	2	**73**	2	1	0	2	1
**LE**	2	1	2	1	0	2	2	1	2	0	2	1	0	2	2	1	0	2	1	2	**69**	2	2	1	0
**HE**	1	2	0	2	1	1	2	2	0	1	1	2	2	1	1	2	1	1	2	0	2	**70**	1	0	2
**CH**	2	0	2	1	2	2	4	0	2	2	1	2	1	2	2	0	2	2	1	2	1	2	**62**	2	1
**HD**	1	2	2	2	1	0	1	2	2	1	2	0	4	1	2	3	1	0	2	1	2	3	2	**61**	2
**NB**	2	2	1	0	4	2	2	3	1	2	0	4	1	2	1	2	0	2	1	2	0	2	1	2	**60**
**Average**	**65.96%**																								

**Table 7:** Performance of Semidefinite Embedding method (instead of using the developed approach) using MRI dataset (Unit %).

**Illnesses**	**FS**	**MN**	**GL**	**VD**	**CA**	**HY**	**CC**	**MA**	**CS**	**MI**	**AD**	**CT**	**ME**	**PD**	**SR**	**AL**	**CJ**	**MB**	**AV**	**MS**	**LE**	**HE**	**CH**	**HD**	**NB**
**FS**	**79**	0	1	0	2	2	0	1	0	2	0	1	2	0	1	0	1	2	0	2	1	2	0	1	0
**MN**	2	**82**	0	1	0	1	0	0	2	0	1	0	0	2	0	1	0	1	2	0	2	0	1	0	2
**GL**	0	1	**87**	0	2	0	1	0	0	1	0	2	1	0	0	0	1	0	0	1	0	2	0	1	0
**VD**	2	0	1	**77**	0	2	1	1	0	2	2	0	1	0	2	0	2	2	1	0	1	0	2	0	1
**CA**	0	1	0	2	**81**	0	2	0	2	0	1	2	0	2	0	1	0	0	1	2	0	1	0	2	0
**HY**	2	0	1	0	1	**79**	0	1	0	2	0	1	2	0	1	2	1	0	2	0	1	0	2	0	2
**CC**	1	2	0	2	0	1	**75**	0	2	1	2	0	1	2	0	1	2	2	0	1	0	2	2	1	0
**MA**	0	1	2	0	2	0	1	**83**	0	0	1	2	0	1	2	0	0	1	2	0	0	1	0	0	1
**CS**	1	0	0	1	0	2	0	1	**85**	0	2	0	1	0	0	2	1	0	0	2	1	0	1	0	0
**MI**	0	2	0	0	1	0	2	0	0	**87**	0	1	0	0	2	0	0	1	0	0	0	2	0	1	1
**AD**	2	0	1	0	0	0	0	2	0	0	**88**	0	2	0	0	0	1	0	2	0	0	0	1	0	1
**CT**	0	1	0	2	1	2	0	0	1	2	0	**80**	0	1	2	1	0	2	0	1	2	0	0	2	0
**ME**	1	0	2	0	0	0	2	1	0	0	1	0	**84**	0	1	0	2	0	1	0	0	2	1	0	2
**PD**	0	2	0	1	0	1	0	0	2	1	0	1	0	**85**	0	2	0	1	0	1	1	0	0	2	0
**SR**	1	0	1	0	2	0	1	2	0	0	2	0	1	0	**82**	0	2	0	1	0	0	2	1	0	2
**AL**	0	2	0	1	0	1	0	1	2	1	0	1	0	1	2	**81**	0	2	0	1	1	0	2	1	0
**CJ**	3	0	0	0	1	1	1	2	2	0	0	0	0	0	1	1	**78**	2	1	1	0	0	2	3	1
**MB**	2	1	1	1	0	2	0	1	1	1	2	1	0	1	0	0	1	**79**	2	2	1	1	0	0	0
**AV**	0	0	0	0	3	0	0	0	0	0	1	1	1	1	1	0	0	2	**89**	0	0	0	0	1	0
**MS**	0	1	0	0	0	2	0	0	0	2	0	0	0	0	0	1	0	1	1	**90**	0	1	0	1	0
**LE**	0	0	0	0	0	0	0	0	2	0	0	1	0	0	1	0	1	0	0	2	**91**	0	1	0	1
**HE**	1	1	1	0	2	1	0	0	0	0	1	2	0	0	0	0	1	1	0	1	1	**86**	0	0	1
**CH**	1	0	0	0	0	2	1	1	1	0	0	0	1	1	1	0	0	1	2	1	0	0	**87**	0	0
**HD**	2	1	0	2	1	0	0	2	0	1	1	0	1	1	2	3	0	0	0	0	1	2	0	**80**	0
**NB**	1	0	1	1	0	1	0	0	1	1	1	2	0	0	0	0	3	1	0	0	0	1	1	0	**85**
**Average**	**83.20%**																								

**Table 8:** Performance of Independent Component Analysis (ICA) method (instead of using the developed approach) using MRI dataset (Unit %).

**Illnesses**	**FS**	**MN**	**GL**	**VD**	**CA**	**HY**	**CC**	**MA**	**CS**	**MI**	**AD**	**CT**	**ME**	**PD**	**SR**	**AL**	**CJ**	**MB**	**AV**	**MS**	**LE**	**HE**	**CH**	**HD**	**NB**
**FS**	**71**	0	2	1	2	2	0	1	2	2	0	1	2	0	2	1	2	0	2	1	1	0	2	2	1
**MN**	1	**77**	0	2	0	0	2	2	1	0	1	2	0	1	2	2	0	1	2	2	0	1	0	1	0
**GL**	2	0	**81**	0	2	1	0	1	0	2	0	1	1	0	1	1	2	0	1	0	2	0	1	0	1
**VD**	0	2	0	**82**	0	1	2	0	1	0	2	0	0	2	0	0	1	2	0	1	0	2	0	2	0
**CA**	1	0	2	1	**73**	0	1	2	1	2	0	1	2	0	2	2	0	1	2	0	2	0	1	2	2
**HY**	0	1	0	0	1	**84**	2	0	2	0	1	2	0	1	0	0	1	0	0	2	0	2	0	1	0
**CC**	2	0	1	1	0	0	**85**	1	0	2	0	0	1	0	0	2	0	0	1	0	1	0	2	0	1
**MA**	0	2	0	2	1	1	0	**81**	1	0	2	1	0	2	1	0	1	2	0	0	0	1	0	2	0
**CS**	1	0	1	0	2	0	2	0	**80**	1	0	2	0	0	2	1	0	0	1	2	2	0	2	1	0
**MI**	0	2	0	1	0	0	0	2	0	**88**	0	0	1	0	0	0	2	1	0	0	0	2	0	0	1
**AD**	2	0	1	0	2	1	2	0	2	0	**74**	1	0	2	1	2	0	0	2	1	1	0	2	2	2
**CT**	2	1	0	2	1	0	2	1	0	2	2	**75**	1	0	2	0	1	2	0	2	0	2	0	2	0
**ME**	0	2	1	0	2	1	1	0	2	1	0	2	**77**	1	0	2	0	1	2	0	1	1	2	0	1
**PD**	1	0	2	1	0	0	2	1	0	0	1	0	2	**79**	1	0	2	2	0	1	2	0	1	2	0
**SR**	2	1	0	0	1	2	0	0	2	1	0	1	0	0	**83**	2	0	0	2	0	0	2	0	1	0
**AL**	0	0	1	1	0	0	1	2	0	0	2	0	2	1	0	**85**	1	0	0	2	0	0	2	0	0
**CJ**	1	0	0	0	2	0	0	0	1	1	0	0	0	0	1	0	**89**	2	0	0	1	0	0	2	0
**MB**	0	2	0	1	0	1	0	2	0	0	0	2	0	0	0	0	0	**90**	0	1	0	0	1	0	0
**AV**	1	0	0	0	0	0	1	0	0	0	2	0	0	1	0	2	0	0	**91**	0	0	2	0	0	0
**MS**	0	0	2	0	1	0	0	0	1	0	0	0	2	0	0	0	0	0	0	**92**	0	0	0	0	2
**LE**	2	1	0	2	0	2	0	1	0	2	2	1	0	0	2	1	2	0	2	1	**76**	0	2	1	0
**HE**	1	0	2	0	1	0	2	0	2	0	1	0	1	2	0	2	0	2	0	2	2	**78**	0	1	1
**CH**	0	2	0	1	0	2	0	1	0	1	0	2	0	2	1	0	1	0	2	0	1	1	**81**	0	2
**HD**	2	0	1	0	2	0	1	0	2	0	2	0	1	0	2	2	0	1	0	1	2	2	0	**77**	2
**NB**	0	1	0	2	0	1	0	2	1	1	0	2	0	1	2	0	2	0	1	2	0	0	2	0	**80**
**Average**	**81.16%**																								

**Table 9:** Performance comparison of the state-of-the-art methods against the proposed approach on MRI brain dataset (Number of utilized images 595 (where, normal = 115, and abnormal = 480)).

Published Methods	Used Methods	Recognition Rates	Misclassification
Orouskhani, et al. [44]	Conditional Deep Triplet Network	92.5%	1.2%
Inglese, et al. [45]	Decision Support System	81.0%	2.5%
Mandle, et al. [46]	Kernel-based SVM	90.2%	3.3%
Abdulmunem, et al. [47]	Deep Belief Network	88.9%	3.5%
Jang, et al. [48]	Sorting Algorithm	72.6%	4.6%
Popuri, et al. [49]	Ensemble Learning	90.3%	3.1%
Latif, et al. [50]	Neural-Network-Based Features with SVM Classifier	89.9%	0.9%
Nawaz, et al. [51]	Multilayer Perception, J48, Meta Bagging, Random Tree	83.8%	2.0%
Assam, et al. [52]	Random Forest	94.1%	3.9%
Islam, et al. [53]	Convolutional Neural Network	78.9%	4.8%
Dehkordi, et al. [54]	Evolutionary Convolutional Neural Network	91.3%	2.0%
Krishna, et al. [55]	Local Linear Radial Basis Function Neural Network	88.7%	3.9%
Takrouni, et al. [56]	Deep Convolutional Network	92.5%	2.0%
Fayaz, et al. [57]	Convolutional Neural Network	86.8%	5.2%
**Proposed Scheme**	**Logistic Regression**	**96.6%**	**3.4%**

**Table 10:** Performance comparison of the state-of-the-art methods and the proposed approach using various evaluation measurements on brain MRI dataset (Number of utilized images 595 (where, normal = 115, and abnormal = 480)).

Published Methods	Used Methods	True Positive	True Negative	False Positive	False Negative
Orouskhani, et al. [44]	Conditional Deep Triplet Network	375	175	60	8
Inglese, et al. [45]	Decision Support System	360	178	57	7
Mandle, et al. [46]	Kernel-based SVM	350	177	63	9
Abdulmunem, et al. [47]	Deep Belief Network	365	178	67	10
Jang, et al. [48]	Sorting Algorithm	380	174	66	8
Popuri, et al. [49]	Ensemble Learning	350	175	61	7
Latif, et al. [50]	Neural-Network-Based Features with SVM Classifier	355	176	64	11
Nawaz, et al. [51]	Multilayer Perception, J48, Meta Bagging, Random Tree	375	169	59	6
Assam, et al. [52]	Random Forest	380	172	58	7
Islam, et al. [53]	Convolutional Neural Network	370	174	60	9
Dehkordi, et al. [54]	Evolutionary Convolutional Neural Network	360	173	55	8
Krishna, et al. [55]	Local Linear Radial Basis Function Neural Network	355	177	62	9
Takrouni, et al. [56]	Deep Convolutional Network	365	171	68	10
Fayaz, et al. [57]	Convolutional Neural Network	370	178	60	8
**Proposed Approach**	**Logistic Regression**	**405**	**185**	**30**	**5**

**Table 11:** Performance comparison of the state-of-the-art methods and the proposed approach using sensitivity, accuracy, and specificity on brain MRI dataset (Number of utilized images 595 (where, normal = 115, and abnormal = 480)).

Published Methods	Used Methods	Sensitivity	Accuracy	Specificity
Orouskhani, et al. [44]	Conditional Deep Triplet Network	93.1%	92.5%	87.8%
Inglese, et al. [45]	Decision Support System	83.8%	81.0%	76.5%
Mandle, et al. [46]	Kernel-based SVM	89.3%	90.2%	92.4%
Abdulmunem, et al. [47]	Deep Belief Network	91.4%	88.9%	85.4%
Jang, et al. [48]	Sorting Algorithm	76.7%	72.6%	67.3%
Popuri, et al. [49]	Ensemble Learning	91.6%	90.3%	86.6%
Latif, et al. [50]	Neural-Network-Based Features with SVM Classifier	90.2%	89.9%	88.1%
Nawaz, et al. [51]	Multilayer Perception, J48, Meta Bagging, Random Tree	81.2%	83.8%	85.3%
Assam, et al. [52]	Random Forest	95.6%	94.1%	89.9%
Islam, et al. [53]	Convolutional Neural Network	80.8%	78.9%	74.0%
Dehkordi, et al. [54]	Evolutionary Convolutional Neural Network	93.6%	91.3%	90.1%
Krishna, et al. [55]	Local Linear Radial Basis Function Neural Network	89.4%	84.1%	85.2%
Takrouni, et al. [56]	Deep Convolutional Network	93.1%	90.5%	87.6%
Fayaz, et al. [57]	Convolutional Neural Network	88.7%	84.9%	90.2%
**Proposed Approach**	**Logistic Regression**	**97.9%**	**96.6%**	**92.1%**

## Data Availability

Not applicable.
